# Supplementary figures and images for: The TOG protein Stu2 is regulated by acetylation
Source: PLoS Genet. 2022 Sep 9;18(9):e1010358. doi: 10.1371/journal.pgen.1010358 (PMC9491610; doi:10.1371/journal.pgen.1010358)

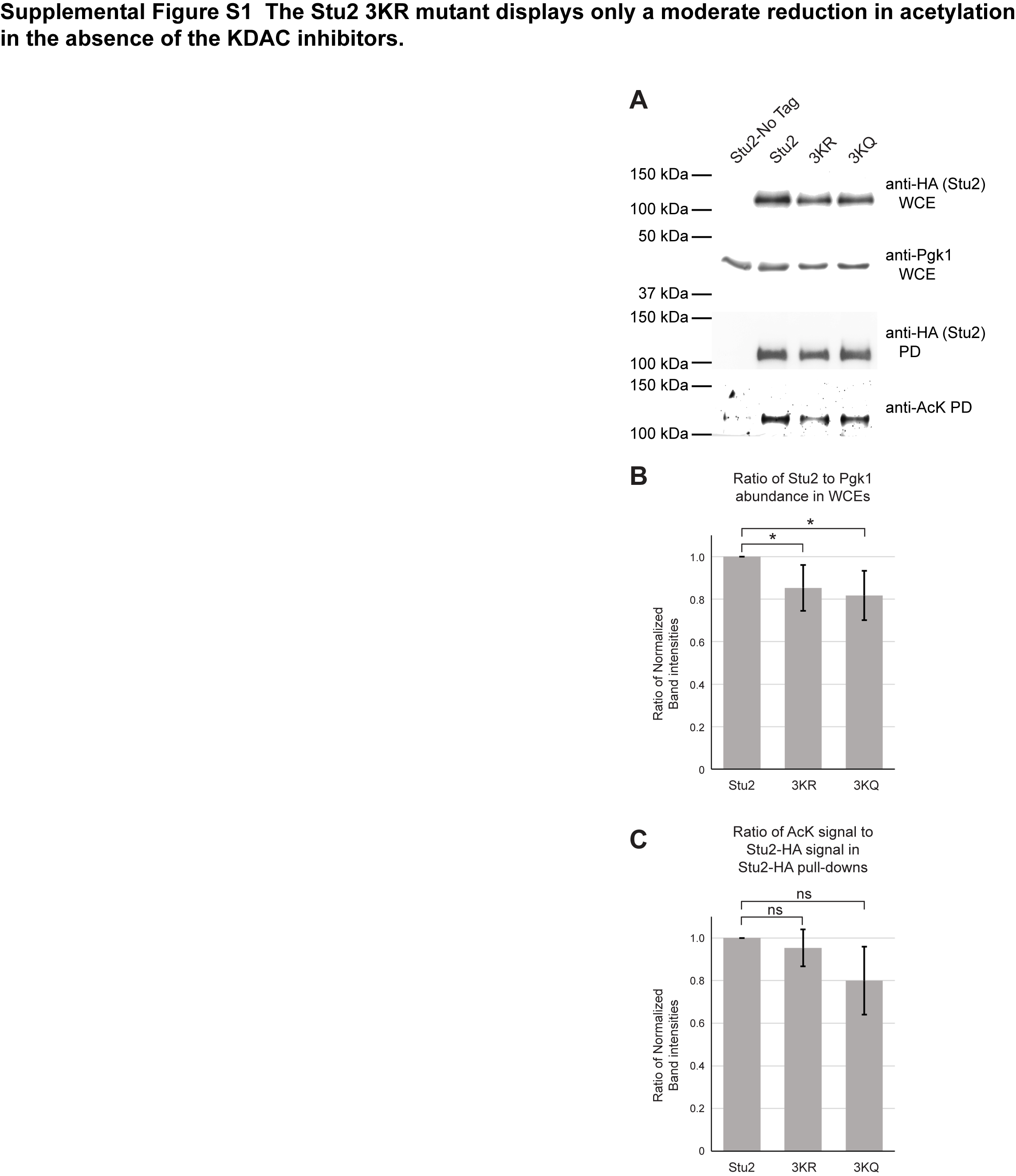

Supplement: S1 Fig — Stu2-3K mutants with mutation of lysines 252, 469, and 870 in HA epitope- tagged strains were analyzed for acetylation by western blot. (A) Cells expressing Stu2-HA (yRM12358), untagged Stu2 (yRM12359), Stu2-3KR-HA (yRM12364), or Stu2-3KQ-HA (yRM12369) were immunoprecipitated and western blotted with anti-HA and anti-AcK to evaluate Stu2 acetylation. Band densitometry was performed to quantify reductions in Stu2 acetylation. (B) The ratio of Stu2 present in WCEs relative to the Pgk1 loading control was evaluated to determine differences in Stu2 steady state abundance from logarithmically growing cell cultures. (C) The ratio of anti-AcK to Stu2-HA signal from immunoprecipitations was determined to evaluate reductions in acetylation for the Stu2-3K mutants compared to wild type. Ratios of Stu2-HA to Pgk1 protein from WCEs and AcK signal to Stu2-HA signal from pulldowns displayed as mean ± SEM from four biological replicates. Student’s t-tests performed on densitometry values prior to normalization to WT controls; * p < 0.1, ** p < 0.05, *** p < 0.01 (raw quantitative data are available in S1 Supplementary file). (TIF) [file pgen.1010358.s001.tif]

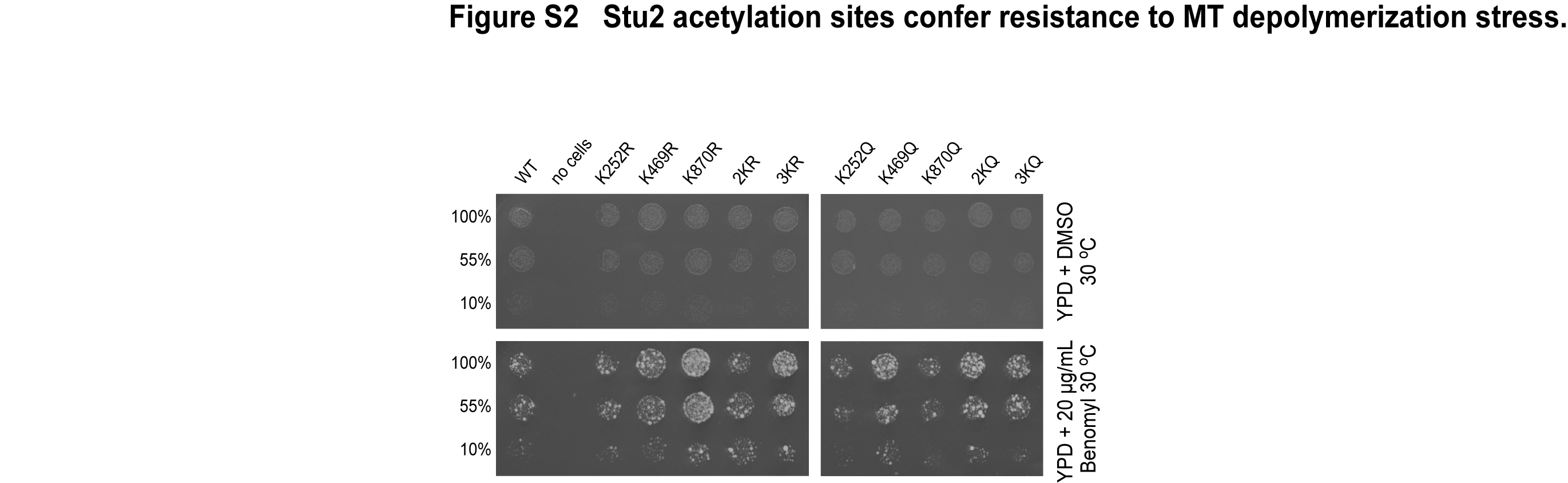

Supplement: S2 Fig — Haploid yeast containing LEU2 plasmids with STU2 (yRM12358), K252R (yRM12360), K469R (12361), K870R (12362), 2KR (yRM12363), 3KR (yRM12364), K252Q (yRM12365), K469Q (yRM12366), K870Q (yRM12367), 2KQ (yRM12368), or 3KQ (yRM12369) were transferred to YPD containing DMSO or DMSO + 20 μg/mL benomyl and grown at 30°C. (TIF) [file pgen.1010358.s002.tif]

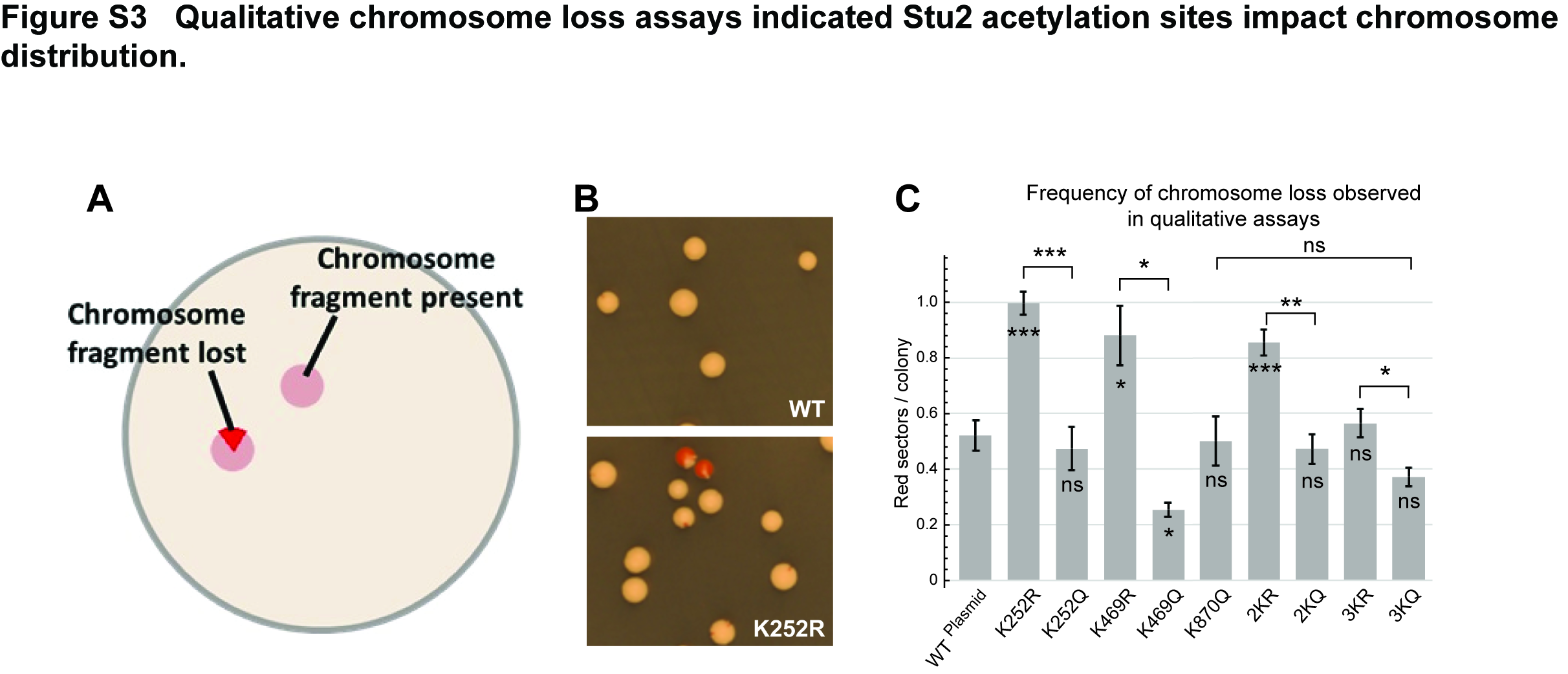

Supplement: S3 Fig — (A) Chromosome transmission fidelity was qualitatively assessed using a color-sectoring colony assay in which the red phenotype is suppressed by SUP11 present on the 125-kb artificial chromosome (pRM11972/pJS2). (B) Colonies with STU2 plasmids (pRM2119) form red sectors less frequently than cells containing K252R plasmids (pRM11481). Yeast in this assay contained plasmids with STU2 (yRM12253), K252R (yRM12261), K469R (yRM12301), 2KR (yRM12266), 3KR (yRM12269), K252Q (yRM12262), K469Q (yRM12260), K870Q (yRM12270), 2KQ (yRM12267), and 3KQ (yRM12268) in a stu2Δ::HIS3 background, except for the WT-genomic control which had an intact STU2 at its genetic locus and an empty vector instead of the STU2 on a plasmid (yRM12300). (C) The total number of sectors counted was divided by the total number of colonies present to determine chromosomal loss rates of mutants relative to WT controls. Qualitative chromosomal loss data displayed as mean ± SEM of triplicate plates; * p < 0.05, ** p < 0.01, *** p < 0.005 (raw qualitative data are available in S1 Supplementary file). (TIF) [file pgen.1010358.s003.tif]

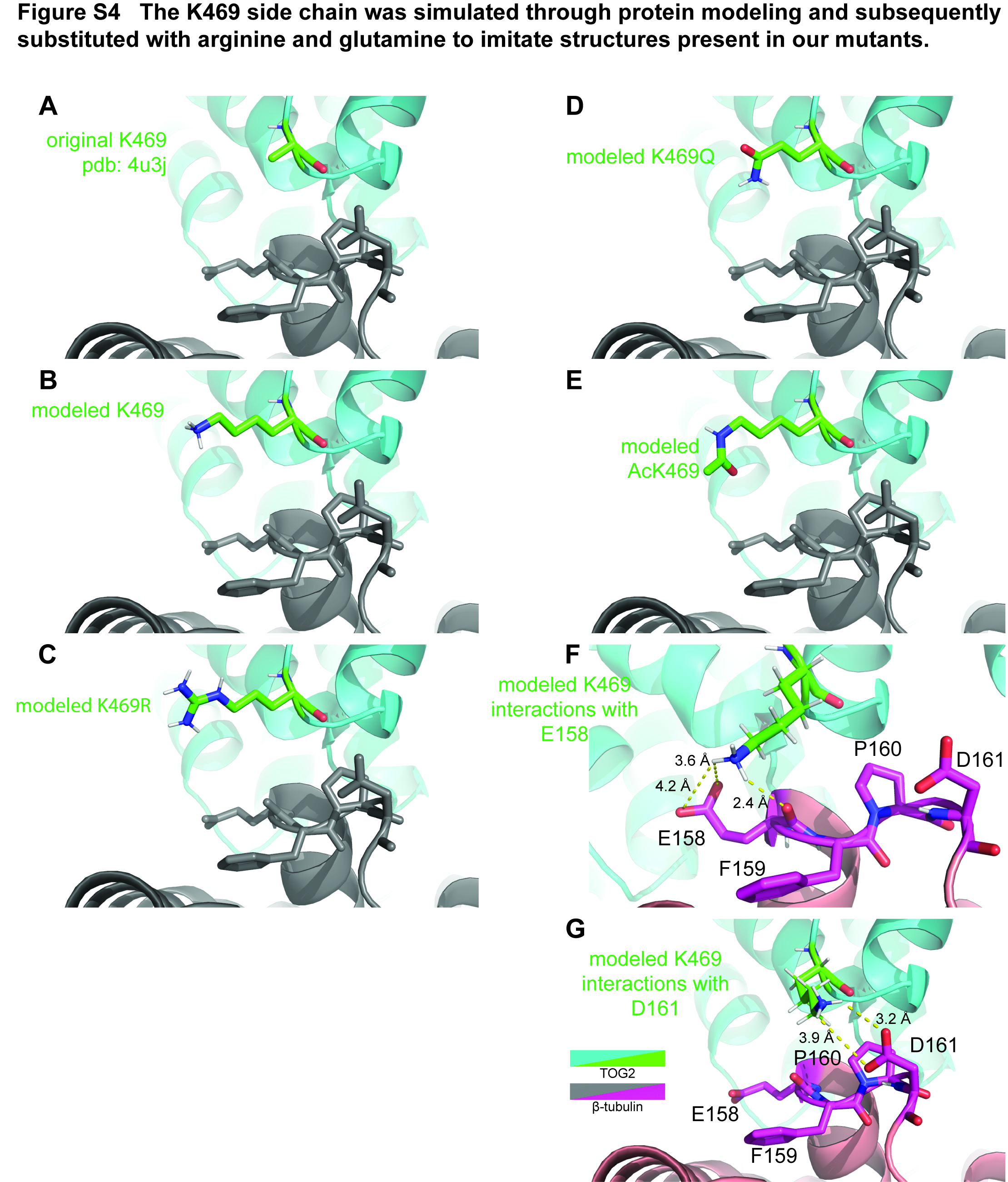

Supplement: S4 Fig — The side chain of K469 was not solved in crystal structures between TOG2 and tubulin heterodimer in pdb:4U3J (A). To gain insight into how K469 may interact with β-tubulin, we modeled the side chain of K469 using PyMOL. The TOG2 K469 side chain is reconstructed in panel B. We modeled side chains for the (C) K-to-R acetylation prevention mutation, the (D) K-to-Q acetylation mimetic mutation, and (E) acetylated K469. Lastly, K469 rotamer confirmations were examined in this model to predict this residue’s interactions with (F) E158 and (G) D161 of β-tubulin. (TIF) [file pgen.1010358.s004.tif]

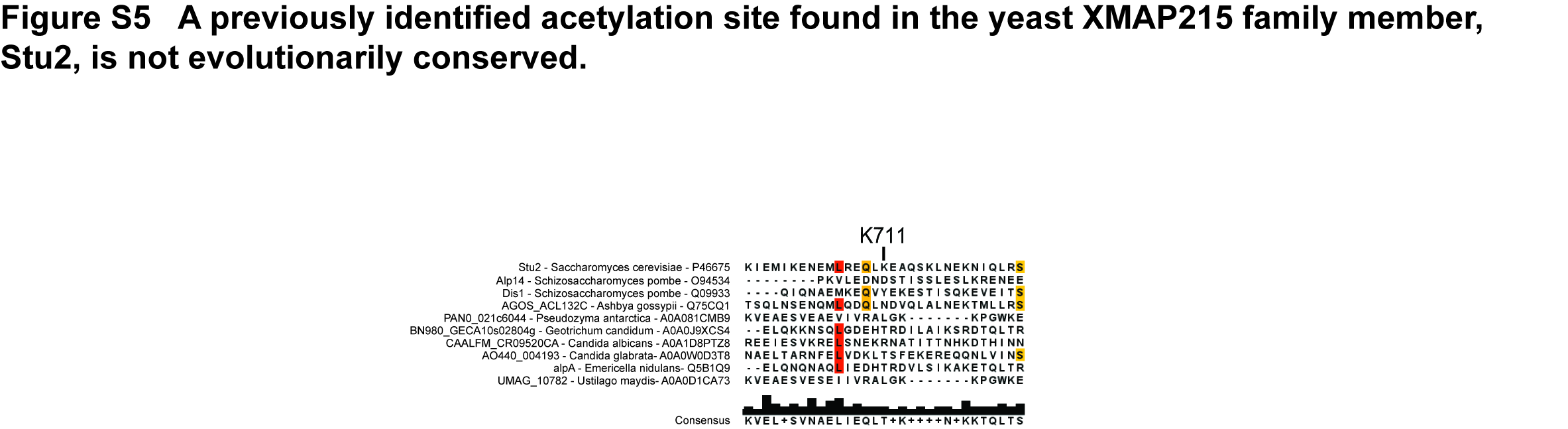

Supplement: S5 Fig — A previous acetylomic screen identified K711 as an acetylated lysine in the yeast XMAP215 family member, Stu2 [74]. To understand its evolutionary conservation, we aligned the K711 with a series of homologues from several fungal species as described above in Fig 3. This analysis suggests that the K711 site is not evolutionarily conserved. (TIF) [file pgen.1010358.s005.tif]

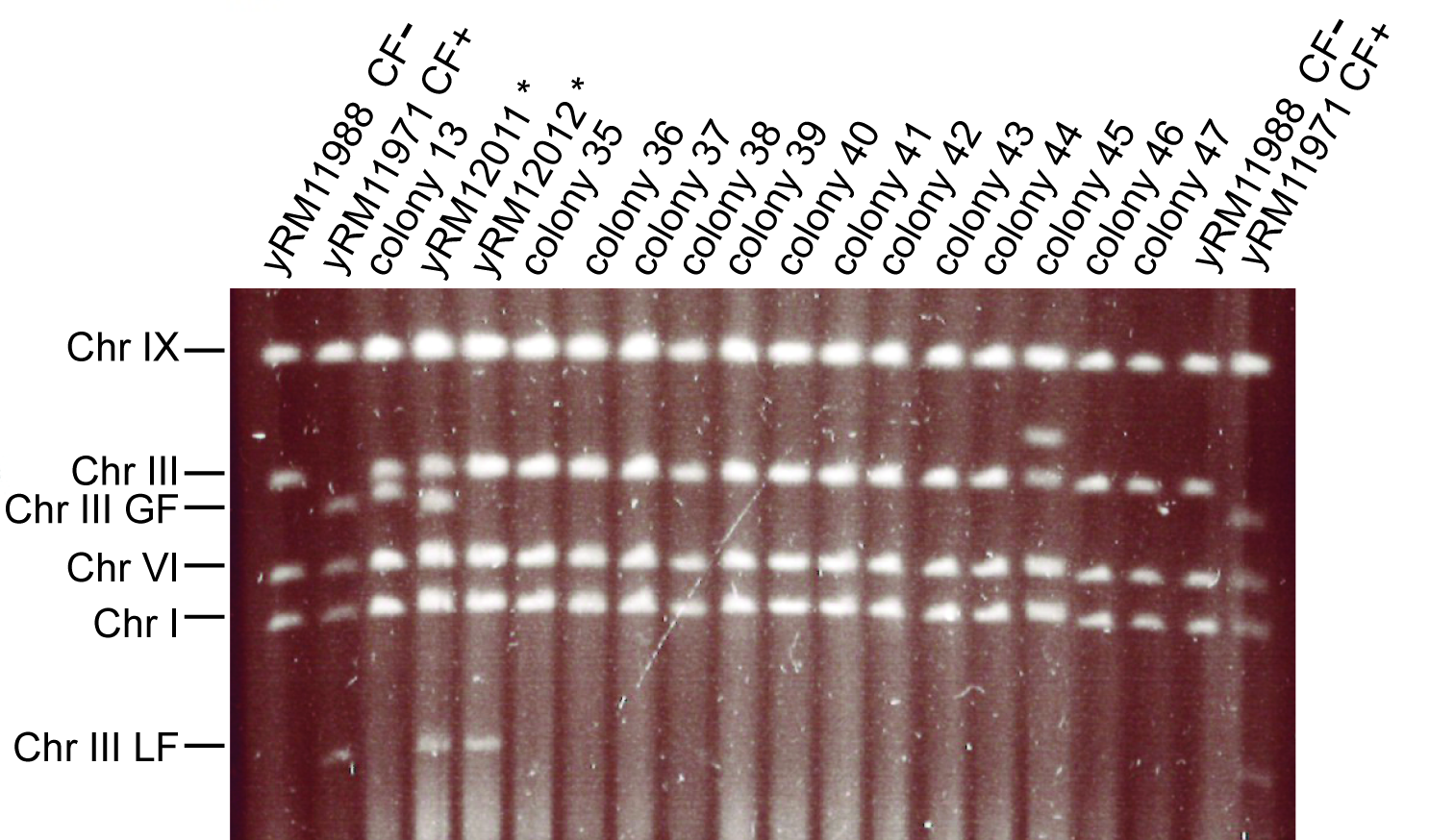

Supplement: S6 Fig — Fragmentation of Chromosome III was generated as described in [33], which contains an illustration of the methods by which we constructed our chromosome loss strain. Briefly, linearization and cross one during pJS2/pRM11972 integration occurs between the γ’ element of the vector and endogenous yeast γ’ sequences to generate a chromosome fragment precursor with a one sided telomeric DNA cap. A second cross occurs in the D8B genetic sequence specific to the left arm of chromosome III to yield a 125-kb Chromosome III fragment. Shero et al (1991) [33] provides a pictoral representation of the creation of this chromosome 3 fragment. (TIF) [file pgen.1010358.s006.tif]

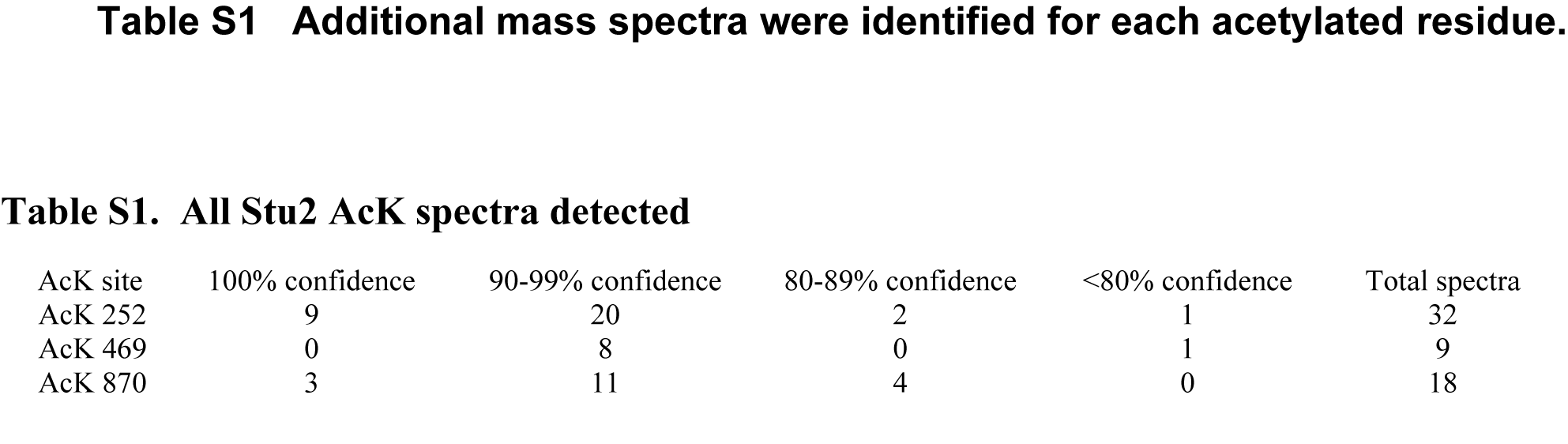

Supplement: S1 Table — Additional mass spectra were identified for each of the acetylated residues in Stu2. Listed are all of the confidence scores associated with each putative AcK specta. AcK 252 had a total of 32 spectra with the following confidence scores: 9 with 100%, 5 with 99%, 4 with 97%, 8 with 96%, 2 with 95%, 1 with 94%, 1 with 83%, 1 with 82%, 1 with 32%. AcK 469 had a total of 10 spectra with the following confidence scores: 1 with 97%, 3 with 96%, 1 with 95%, 1 with 94%, 1 with 93%, 1 with 91%, and 1 with 70%. AcK 870 had a total of 18 spectra with the following confidence scores: 3 with 100%, 4 with 99%, 3 with 98%, 3 with 97%, 1 with 96%, 1 with 86%, 1 with 85%, 1 with 82%, and 1 with 80%. All spectra fell well within a 30 ppm mass accuracy with the vast majority of parent ion masses deviating less than 3 ppm. (TIF) [file pgen.1010358.s007.tif]

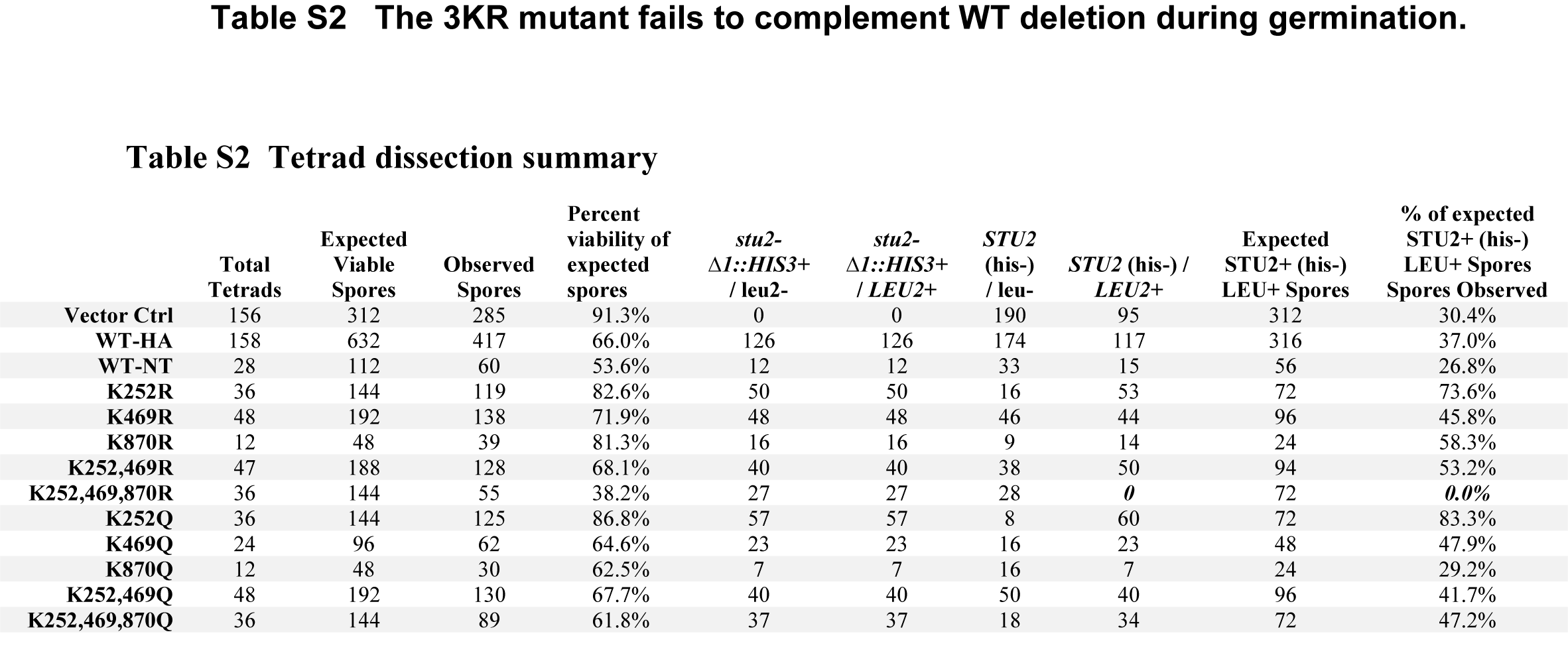

Supplement: S2 Table — A STU2/stu2-Δ1::HIS3 heterozygous diploid (yRM11105) was transformed with wild-type STU2-HA (pRM2119), empty vector (pRM2200), untagged wild-type STU2 (pRM6507), K252R (pRM11481), K469R (pRM11249), K870R (pRM12016), 2KR (pRM11966), 3KR (pRM11976), K252Q (pRM11482), K469Q (pRM11254), K870Q (pRM12015), 2KQ (pRM11969), and 3KQ (pRM11974). Each strain was then sporulated and tetrads dissected. STU2 deletion was marked with HIS3 (stu2-Δ1::HIS3). Yeast centromeric plasmids expressing STU2 or stu2 mutants were marked with a LEU2 gene. In this analysis, the total tetrads for each dissected strain are reported. The expected viable spores reflect the 2:2 nature of sister chromatid distribution as well as the inability of our vector control to rescue stu2 deletion. Observed spores represents the total number of spores that grew. The four categories of possible genotypes are reported in columns 5–8. Notably, the K252,469,870R (3KR) mutant fails to produce viable spores in the presence of an endogenous copy of STU2. In the absence of endogenous STU2 (stu2-Δ1::HIS3), spores containing the 3KR mutant grow. (TIF) [file pgen.1010358.s008.tif]
